# Aggression Against Police Officers and Behavior Toward Citizens: Reciprocal Influence or Common Causes?

**DOI:** 10.3389/fpsyg.2022.866923

**Published:** 2022-06-23

**Authors:** Lisa van Reemst, Tamar Fischer, Frank Weerman

**Affiliations:** ^1^Department of Criminal Law and Criminology, Faculty of Law, Vrije Universiteit Amsterdam, Amsterdam, Netherlands; ^2^Netherlands Institute for the Study of Crime and Law Enforcement (NSCR), Amsterdam, Netherlands; ^3^Department of Criminology, Erasmus School of Law, Erasmus Universiteit Rotterdam (EUR), Rotterdam, Netherlands

**Keywords:** police, victimization, violence, use of force, victim-offender overlap, aggression, conflict

## Abstract

Police officers are often the victim of aggression by citizens, which has negative consequences for them and society in general. Therefore, it is important to gain knowledge about related factors. This study examines to what extent experiencing aggression as a police officer is related to their patience with citizens and use of force weapons and tools. Two explanations based on the victim-offender overlap are examined: experiences of aggression and behavior toward citizens influence each other vs. both have common causes. This study was conducted on the basis of a longitudinal survey among police officers (*N* = 693). The results show that behavior toward citizens, including patience with citizens and the use of force is related to the aggression police officers experience. The association decreases but does not disappear when common causes are taken into account, in this case socio-demographic and work-related characteristics. No direct support is found for reciprocal influence. Implications and suggestions for further research are described.

## Introduction

Police officers are relatively often confronted with aggression by citizens (Van Reemst and Fischer, 2019). This may involve verbal or physical violence, (sexual) intimidation or discrimination. Previous research has shown that 88 percent of police officers experienced psychological aggression (verbal, intimidation, and discrimination) and that 61 percent experienced physical violence in 1 year (Van Reemst and Fischer, 2019). It is not surprising that police officers are often confronted with this behavior, taken into account the frequent contact with citizens and the nature of that contact. Although the registered numbers of aggression against the police do not show a clear increase in recent years, they also do not show a decrease (for a literature review in the Netherlands, Aarten et al., 2020; Police, 2021). In 2020, 12,543 reports of violence against police officers had been registered with the police in the Netherlands (Police, 2021). Concerns are regularly expressed in the media and in policy programs about aggression against the police (Rijksoverheid, 2021). These concerns are fueled by research that has found that experiences of aggression can have a negative impact on employees, such as developing post-traumatic stress or burnout symptoms (Ellrich, 2016; Ellrich and Baier, 2017). In addition, aggression against the police appears to be unequally distributed: some police officers experience more aggression than others. For example, a tenth of the police employees experience more than half of the violent incidents in a year (Fischer and Van Reemst, 2014; Van Reemst, 2016). It is therefore important to gain insight into factors that increase or decrease the risk of aggression against police officers.

This study examines the relationship between aggression against police officers and their own behavior toward citizens. The police have the authority to use (appropriate) force through the so-called monopoly of violence. Rules and conditions for this are described in the Police Act and official instructions (Timmer, 2005; Timmer and Visser, 2014). In practice, the use of force by the Dutch police usually involves physical violence (69%), use of pepper spray (18%), holding or use a firearm (15%) and a baton (7%) (Kuin et al., 2020). Police officers will sometimes actually have to use force, for example to protect others and themselves. But usually, police officers can resolve situations and conflicts without violence, for example by talking or using verbal urges.

However, each officer will behave differently toward citizens. Not only will this be visible in the use of force by an officer in his or her career, but also in the patience with citizens. This may be caused by the nature and context of the work situations, personal characteristics, and previous experiences with aggression by citizens.

The central question of this study is: *How are police officers’ experiences of aggression related to their patience with citizens and the use of force weapons and tools?* We not only examine whether there is a relation, but also whether they are causally related and the extent to which the relation can be traced back to common factors.

Our study was informed by literature that focuses on the relationship between victimization and perpetration in the general population, in else, victim-offender overlap literature (for a literature review, see Jennings et al., 2012). This body of research suggest various explanations and therefore helps to think about the potential relationship between officers’ experiences of aggression (or “victimization”) and differences in behavior toward citizens. However, it is important to emphasize that we do not consider any use of force by officers as “perpetrating” or “offending.” These labels do not apply to the context of police work where the use of force, including means of force, are legitimate, as discussed above. Moreover, impatience with citizens, also included in this study, is no use of force at all. The literature on victimization and perpetration only serves to theorize about the underlying processes of the relationship between experiences of aggression and behavior toward citizens.

### Theoretical Framework and Literature Review

The victim-offender overlap literature indicates that there are people who are both victims and perpetrators of crime in their lives (Jennings et al., 2010; Ousey et al., 2011; Jennings et al., 2012; Tillyer and Wright, 2014). There are two main general explanations^1^ for this, which may also be important for the relationship between police officers’ experiences of aggression and their behavior toward citizens. One of the general explanations assumes that there is a causal relationship between the two characteristics, which either means that one influences the other or that there is a reciprocal influence. The other explanation assumes that the relationship between the two characteristics can be traced back to other characteristics, in other words that victimization and perpetration have common causes (Jennings et al., 2012; Rokven, 2016). These explanations are not mutually exclusive, however: it is possible that both explanations apply.

On the one hand, a causal relationship can mean that being a perpetrator of crime leads to becoming a victim more often, for example through reprisals by the victim or bystanders. On the other hand, being a victim of crime can encourage someone to become a perpetrator of crime, for example by taking revenge or develop a more aggressive response pattern.^2^ In this way, causal relationships can appear in both directions. As discussed, the current study does not investigate perpetration of crimes, but impatience with citizens and the use of force among police officers. However, the causal relationships could also apply to these behaviors among police officers. Applied to the police and the relationship between experiences of aggression and behavior toward citizens, a causal relationship would mean that police officers become more impatient toward citizens through experiencing aggression, or that they experience aggression more often because of their (im) patience.

The idea that people with certain behavioral characteristics, such as being impatient, experience aggression more often is not new. Indeed, victimology started with research into “victim precipitation,” the (active) role of the victim in the formation of victimhood (Von Hentig, 1948; Wolfgang, 1958; Amir, 1971). However, this type of research was soon considered to blame the victim. In victimology, researchers then often studied characteristics of the perpetrator or the situation of violent situations. Nevertheless, later in time it was recognized that it is also important to study the psychological and behavioral characteristics of the victim (Finkelhor and Asdigian, 1996; Van Reemst, 2016). In addition, in studies about workplace violence, it is argued that employee characteristics, such as being impatient or having a more aggressive attitude, can cause others with whom employees come into contact to feel their personal norms violated, which in turn can lead to aggression (Aquino and Thau, 2009; Bowling et al., 2010).

In previous research, support has been found for the notion that people who experience aggression are also more impatient themselves or use violence. For example, people would react more aggressively immediately after an aggression experience (Andersson and Pearson, 1999; Hershcovis and Reich, 2013), possibly to restore feelings of injustice. Psychological characteristics could also change after experiencing aggression in the work situation, after which employees might behave differently. For example, it has been found that employees experience more negative affect, or negative feelings, after experiencing mild forms of aggression (Giumetti et al., 2013). Existing research therefore suggests that a causal relationship between behavior toward people and the experience of aggression can work in both directions, but this has only been investigated to a limited extent among police officers.

The alternative explanation for the victim-offender overlap implies that characteristics of the situation or of a person cause that person to become both victim and perpetrator. For example, undertaking risky activities or living in deprived neighborhoods can make someone more likely to experience aggression as well as to be the perpetrator. This stems from opportunity theories that are sometimes applied to victim-offender overlap (Schreck et al., 2008). Previous research on the victim-offender overlap in the general population found that half of the relationship between victimization and perpetration could be explained by socio-demographic characteristics and previous experiences with crime (Rokven et al., 2013). This statement could also apply to impatience and the use of force among police officers, instead of perpetration. Characteristics of police officers that could explain experiences of aggression, as well as impatience and use of force, are socio-demographic characteristics such as age and gender. In addition, work-related characteristics such as the region in which officers work, the type of services they perform, or how often they have contact with citizens could explain experiences of aggression. For example, in interviews with emergency responders, respondents also indicated that both their behavior and the risk of experiencing aggression differ between work locations (Van Reemst, 2020). Therefore, in this explanation there is not a direct link between victimization (the experiences of aggression) and behavior toward citizens, but there are other characteristics that predict both.

Based on the theories and studies discussed, this study expects that police officers’ experiences of aggression are related to their patience with citizens and the use of force weapons and tools. So far, little research has been conducted on the relationship between police officers’ experiences of aggression and the way they behave toward citizens in the Netherlands. In a study among police officers in Belgium, it was found that more frequent use of force is associated with more frequent experience of aggression by citizens (Noppe, 2018). An indirect indication of the link is presented in a previous study in the Netherlands, which showed that experiences of aggression are associated with how police officers mentally process and respond to work situations, also referred to as “social information processing” (Van Reemst et al., 2015). There are also studies in other countries that indicate that there is a relationship between the experience of aggression and characteristics that may be related to the behavior toward citizens, including psychological characteristics of the officer (e.g., Ellrich and Baier, 2016).

Other variables associated with confrontations with aggression on the one hand and behavior toward citizens on the other have been studied more often. For example, experiences of aggression among police officers appear to be associated with more contact with citizens, more frequent evening and night work, and more frequent contact on the street or in public space (Van Reemst and Fischer, 2019). Employees who work in areas where there is more violence, more family and economic problems, more ethnic inequality and who have a higher population density are also at higher risk of experiencing aggression (Jacobs and Carmichael, 2002; Kaminski et al., 2003; Rabe-Hemp and Schuck, 2007; Barrick et al., 2014). Moreover, younger employees are said to be at higher risk of experiencing aggression (Van Reemst and Fischer, 2019). The use of force by the police in its turn, appears to be related to situational characteristics, such as the region (Kuin et al., 2020; Paoline et al., 2021). Police force appears to be most often used during weekends, at night and in public spaces (Kuin et al., 2020). The use of force also appears to be associated with greater moral support for the use of force (Noppe, 2016). Moreover, force appears to be used more often by male employees (Crawford and Burns, 2008; Kuin et al., 2020) and by employees with less education and less experience (Paoline and Terrill, 2007).

To date, no study has examined the usefulness of the two explanations discussed above for the relationship between experiences of aggression and behavior toward citizens. In this current study, we try to gain more insight into this relationship, although strictly speaking causality cannot be definitively established with (observational) questionnaire research. Based on the literature on victim-offender overlap and the available studies on aggression against the police, we expect that both explanations will hold to a certain extent. Police officers would then develop more impatience toward citizens after experiencing aggression and vice versa, if they are more impatient, they would experience aggression more often. In addition, the relationship between experiences of aggression and the use of force and impatience would then partly be explained by other factors, more specifically work-related and socio-demographic characteristics.

## Materials and Methods

### Research Design and Sample

This study uses a longitudinal questionnaire study that started in 2015.^3^ The data collection included three measurements, with time intervals of 6 months in between. The first two measurements were used for the analysis in this article. The third measurement is not included in this article, as it is not necessary to answer the research question, and a relatively large number of respondents dropped out in that measurement. The National Police gave permission to send the questionnaires to three (out of ten) regional units in the Netherlands, which differed in urbanization. An employee of the National Police provided the random sample of 2,250 police employees in these units from all employees in primary policing (“Basisteams” in the Netherlands) and sent the emails with the (individual) questionnaire link to the employees on behalf of the researchers. The researchers did not know the contact details of employees, but they gave them a randomly generated code to link their repeated questionnaires. At the start of the questionnaire, respondents were informed about the research, that results were processed anonymously, that they could stop the research at any time and that they could continue again if they wanted to *via* the individual link. Police officers received no incentives for their participation and the total questionnaire took approximately 25 min to complete. At the end of the questionnaire, they were asked whether they wanted to participate in the follow-up questionnaires. In that case, they received a questionnaire with the same questions after 6 and 12 months.

Of the 868 police officers who started the first questionnaire, 606 police officers indicated that they mainly work in primary policing (emergency assistance or as community police officers). Another 102 police officers indicated that they had a different main task, but that they regularly experienced incidents related to emergency assistance (30 per month or more). They were selected for the study. Police officers who indicated that they did not work for whatever reason during the surveyed period were excluded (*N* = 15). This made it sufficiently certain that the 693 respondents had regular contact with citizens. Not every respondent answered all of the relevant questions: In the first measurement, 522 police officers answered all of the relevant questions. In the second measurement, 258 police officers started and 217 police officers completed all relevant questions. Drop-out occurs regularly in longitudinal research designs and this is taken into account in the analysis, see the analytical strategy.

Respondents were on average 39 years old (*M* = 39.03, *SD* = 11.17), 21.1 percent of the respondents were female and 64.8 percent had completed at most (higher or lower) vocational education or lower secondary education (N of which this is known = 676). Respondents had an average of 16 years of work experience (*M* = 15.75, *SD* = 11.12, *N* = 680). In this study, relatively fewer women (21.1 vs. 33.4%, *p* < 0.001) and more younger and fewer older employees (up to and including 34 years of age 42.8 vs. 37.8%; 35–44 years 24.3 vs. 23.3%; 45–54 years 20.9 vs. 20.1%; over 55 years 12.1 vs. 18.9%, *p* < 0.001) participated compared to employees in primary policing in these units.^4^ The ratio of ranks also differed, but not in a specific direction (up to police patrol officers 6.6 vs. 12.6%; constable 11.3 vs. 7.6%; senior constable 36.8 vs. 41.0%; sergeant 39.0 vs. 29.6% and inspector 6.3 vs. 9.2%, *p* < 0.001). These are weak to moderate differences.

### Measurements

Experiences of aggression were measured by asking how often police officers were confronted with 23 different behaviors of citizens in the previous 6 months, ranging from verbal and non-verbal aggression (e.g., gestures), threats and physical violence, with answering options ranging from 0 to 6: never (0), 1 time (1), 2 times (2), 3–5 times (3), 6–10 times (4), 11–20 times (5) and more than 20 times (6).^5^ This instrument is based on research by Dupré et al. (2014) on experiences of aggression by citizens. In these questions, behaviors such as rude behavior, obscene gestures, threats of hitting and (actual) kicking of citizens are addressed. The reliability analyses indicated that these items formed a good scale (α = 0.94 on measure 1, α = 0.95 on measure 2). Because the answer categories are of ordinal measurement level, it was checked in previous research whether they could be considered of interval level. This turned out to yield the same results (Van Reemst and Jongerling, 2021). It is therefore chosen to average the items to create a scale of experiences of aggression.

Impatience with citizens was measured with the six-statement scale developed by Tummers and Musheno (2015). The scale contains statements such as “I lose my temper when I work with citizens” and “Even when citizens become aggressive, I remain calm,” with answering options ranging from 1 to 5: never (1) to always (5). This scale turned out to be sufficiently reliable (α = 0.74 measurement 1, α = 0.77 measurement 2), and answers to statements were averaged. In only the first measurement, questions were asked about the use of force weapons and tools in the career, namely pepper spray, the short baton, the long baton and the service weapon. Answering options ranged from 1 to 5: never, but I was trained (1) to 3 times or more per year (5). A few respondents indicated that they had not been trained for use of force weapons and tools. They were given a missing value for this question. This scale turned out to be sufficiently reliable (α = 0.75). The average of the answers to the questions was calculated if at least three questions were answered. This was chosen because the long baton is not available in all units in the Netherlands. This measure does not include the use of physical violence unrelated to force weapons and tools.

Finally, questions are included about various socio-demographic and work characteristics that could be associated with risky work situations and in this way provide alternative explanations for the relationship between aggression against police officers and behavior toward citizens. Gender, age and education are included as socio-demographic characteristics. For education, “lower vocational education” includes the Dutch “MBO” levels 1 and 2, and “higher vocational education” includes levels 3 and 4. The Dutch university of applied sciences (“HBO”) and research universities (“WO”) have been combined to “higher education” because of a small number of respondents with a completed research university education (*N* = 3). As part of work characteristics, the municipality in which police officers work, their main task (e.g., emergency assistance) and their rank were asked. The municipality has been recoded according to population density based on data from Statistics Netherlands (CBS, 2014). No rank, the rank of aspirant and the rank of patrol officer were combined in relation to a small number of respondents with no rank or with the rank of patrol officer (*N* = 2 and *N* = 4, respectively). Work experience was also surveyed but not included in the analyses because it appeared to be strongly related to age (*r* = 0.88, *p* < 0.001). Nineteen questions of the Risk for Violence scale (LeBlanc and Kelloway, 2002) were used as a final work characteristic. This scale contains work characteristics such as how often people work in the evening or at night, how often people come to their homes, how often people work with people who are under the influence or who have psychiatric problems.^6^ This was questioned separately for full-time and part-time employees and combined so that answering categories mean that this never occurs (1) to five times or more per shift (6). This scale turned out to be reliable (α = 0.87) and the average was calculated. This scale indicates the extent to which there is direct opportunity to experience aggressive situations (LeBlanc and Kelloway, 2002).^7^

### Analytical Strategy

The data analysis was performed in SPSS 27. Due to the dropout rate of respondents within and between the surveys, there may be a selective sample of respondents who have filled in all variables. It was therefore decided to correct for this in the analysis by using multiple imputation (Rubin, 2004). In the event of missing values, the value is estimated based on the variables that have been completed. This thus provides a better estimation of associations. For the multiple imputation model, all variables were included in this study. Thirty imputations were used, which were then pooled. The pooled results are described below in terms of relationships (correlations and regression analyses). Model features of regression analysis are not pooled by SPSS. That is why the average has been calculated for the model characteristics out of the thirty imputations. Only when describing the variables, the values are given without imputation, to reflect what has actually been filled in in the questionnaire.

After describing the variables, the first analysis investigates the cross-sectional relationship between experiences of aggression and the use of force and impatience with citizens. To investigate this relationship, correlations are calculated, and stepwise linear regression analyses are conducted with the use of force weapons and tools as a dependent variable. It is first checked whether there was no multicollinearity based on the VIF values. In the first step of the regression analysis, the variable “experiences of aggression” is the only independent variable. In the second step, socio-demographic and work characteristics are also included. The same is done for the relationship between experiences of aggression and impatience with citizens. If the (expected) relationship between experiences of aggression and use of force and impatience with citizens disappears by adding work and socio-demographic characteristics, the second explanation is supported, that is that other characteristics explain impatience with citizens as well as experiences of aggression. This analysis does not investigate the direction of a possible relationship, but only to what extent a (cross-sectional) relationship between experiences of aggression and behavior toward citizens exists, and to what extent this relationship is explained by other characteristics.

In addition, the relationship between experiences of aggression and impatience with citizens is longitudinally investigated, testing both possible directions, from experiences of aggression to impatience with citizens, and from impatience with citizens to experiences of aggression. This analysis is only performed with impatience with citizens and not with the use of force, because this variable was not measured at T2. After calculating bivariate correlations, we estimated a cross-lagged panel model (Kenny, 1975) in R, using Full Information Maximum Likelihood (FIML) to use all available information (as an alternative to multiple imputation). The R package “lavaan” is used for this (Rosseel, 2012). Only respondents who have completed at least one of the variables in the model are included in the analysis. In this model, the association between experiences of aggression T1, experiences of aggression T2, impatience with citizens T1 and impatience with citizens T2 is estimated simultaneously. If a longitudinal relationship is found between impatience with citizens and experiences of aggression (from T1 to T2), this supports the first explanation, namely a possible causal relationship. In this way we can draw conclusions about the possible direction of the association with some certainty, but it cannot be ruled out that alternative explanations are overlooked. An experimental design is needed for definitive conclusions about causality.

## Results

**FIGURE 1:**
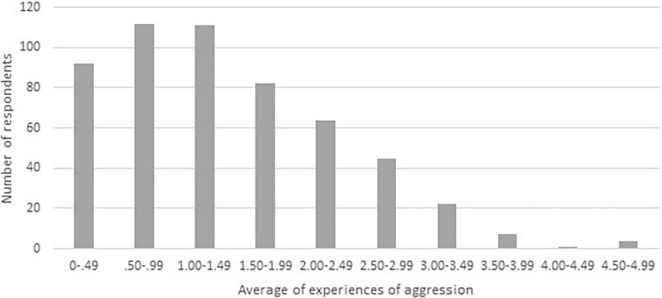
Distribution of experiences of aggression (measured on a scale from 0 to 6) of police officers (*N* = 540).

Respondents differed in how much aggression they had experienced in the previous 6 months. Some never experienced aggression (answer category 0, on average over all items), while others experienced the different behaviors of citizens on average around 11–20 times (answer category 5). This is made visible in Figure 1, for which the experiences of aggression are divided into categories. On average, the different behaviors were experienced between once and twice in the previous 6 months (*M* = 1.42, *SD* = 0.93).

Furthermore, the police officers were on average “rarely” impatient (*M* = 2.05, *SD* = 0.43) and used violence between less than once every 5 years (answer category 2) and 1–4 times every 5 years (answer category 3), although differences appear to exist between respondents (*M* = 2.56, *SD* = 1.09). This is shown in Table 1, which also shows the descriptive data for the other relevant variables.

**TABLE 1:** Descriptive values of variables in the study.

		%	*M*	*SD*	*N*

Aggression experience and behavior toward citizens					
Experiences of aggression T1 (0–6)			1.42	0.93	540
Impatience T1 (1–5)			2.05	0.43	529
Use of force weapons and tools T1 (1–5)			2.56	1.09	522
**Socio-demographic characteristics**					
Gender	*Male*	78.4			530
	*Female*	21.6			146
	*Total*	100			676
Age (20–63)			39.03	11.17	676
Education (highest completed)	*Lower secondary education or lower vocational education*	15.4			104
	*Higher vocational education*	49.4			334
	*Higher secondary education*	23.7			160
	*Higher education*	11.5			78
	*Total*	100			676
**Work characteristics**					
Population density of the area (44–5309)			2040.98	1139.09	693
Main job	*Emergency assistance*	65.1			451
	*Community officer*	20.8			144
	*None of the above*	14.1			98
	*Total*	100			693
Rank	*Up to police patrol officer*	6.6			46
	*Constable*	11.3			78
	*Senior constable*	36.8			255
	*Sergeant*	39.0			270
	*Inspector*	6.3			44
	*Total*	100			693
Risk for violence (1–6)			3.54	0.68	680

Table 2 shows the bivariate relationships in T1. This table shows that the experiences of aggression of police officers in the past 6 months are weakly to moderately related to impatience with citizens (*r* = 0.29, *p* < 0.001) and strongly related to the use of force in the career (*r* = 0.53, *p* < 0.001). Experiencing aggression is therefore related to the way police officers behave toward citizens.

**TABLE 2:** Bivariate correlations of dichotomous, ordinal and interval variables T1, based on pooled results using multiple imputation (*N* = 693)^a^.

	1	2	3	4	5	6	7	8
1. Experiences of aggression (0–6)	–							
2. Impatience (1–5)	0.29***	–						
3. Use of force (1–5)	0.53***	0.23***	–					
4. Gender (1/2, 2 = female)	−0.16***	0.08^+^	−0.11*	–				
5. Age (20–63)	−0.21***	−0.11*	−0.12**	−0.18***	–			
6. Education (1–4)	0.01	–0.03	–0.00	0.06	−0.08*	–		
7. Population density (44–5,309)	0.16***	0.06	0.21***	0.01	–0.05	0.09*	–	
8. Rank (1–5)	−0.09*	–0.03	0.07^+^	−0.08*	0.57***	0.11**	0.02	–
9. Risk for violence (1–6)	0.63***	0.23***	0.55***	−0.08*	−0.13***	0.03	0.11**	0.06

***p < 0.001, **p < 0.01, *p < 0.05, ^+^p < 0.10.

*^a^Without multiple imputation, this analysis would contain data from 522 respondents. The results with multiple imputation differ in significance from results without multiple imputation for only four correlations: the relationship between rank and experiences of aggression, and between education and age, were not significant without multiple imputation (p > 0.05). The relationship between rank and use of force, and between rank and risk for violence, was significant without multiple imputation. The results do not differ in the direction of significant associations.*

Table 3 shows the results of the linear regression analyses with the two dependent variables relating to behavior toward citizens, use of force weapons or tools and impatience with citizens. There was no evidence of multicollinearity based on the Variance Inflation Factor (VIF) values. Model 1 once again shows the bivariate relationship between experiences of aggression and behavior toward citizens. In the second model, socio-demographic characteristics and work characteristics are added. By adding these, the relationship between experiences of aggression and behavior toward citizens becomes weaker, as can be seen from the lower B coefficients of aggression experience in model 2 compared to model 1. For the use of force, the B coefficient decreases by 46 percent, for impatience with citizens by 15 percent. However, the associations between experiences of aggression and behavior toward citizens remain significant (*p* < 0.001). This means that the socio-demographic and work characteristics do not fully explain the relationship.

**TABLE 3:** Regression models of the associations between experiences of aggression and behavior toward citizens, adding socio demographic and work characteristics T1, based on pooled results using multiple imputation (*N* = 693)^a^.

	Dependent variable: use of force weapons/tools						Dependent variable: impatience with citizens					
	Model 1			Model 2			Model 1			Model 2		
	*B*	*SE*	*p*	*B*	*SE*	*p*	*B*	*SE*	*p*	*B*	*SE*	*p*
Experiences of aggression (0–6)	0.61	0.05	**< 0.001**	0.33	0.05	**< 0.001**	0.13	0.02	**< 0.001**	0.11	0.03	**< 0.001**
Gender (1/2, 2 = female)				–0.10	0.10	0.33				0.13	0.05	**0.006**
Age (20–63)				–0.01	0.01	0.10				–0.00	0.00	0.61
Education (1–4)				–0.06	0.04	0.18				–0.02	0.02	0.29
Population density (44–5,309)				0.00	0.00	**< 0.001**				0.00	0.00	0.75
Main job (none of jobs below = reference category)												
Emergency assistance				0.19	0.12	0.11				0.10	0.05	0.07
Community police officer				–0.03	0.14	0.86				0.03	0.06	0.69
Rank (1–5)				0.18	0.05	**0.001**				0.02	0.02	0.42
Risk for violence (1–6)				0.53	0.09	**< 0.001**				0.04	0.04	0.28
Constant	1.74	0.08		–0.11	0.36		1.88	0.04		1.54	0.16	
**Model characteristics**												
*F-value, p*	276.47	< 0.001		49.87	< 0.001		64.11	< 0.001		10.19	< 0.001	
*df regression and residual*	1	691		9	683		1	691		9	683	
*Adjusted R* ^2^	0.28			0.39			0.08			0.11		

*^a^Without multiple imputation, the model for predicting use of force would contain data from 522 respondents and that predicting impatience from 529 respondents. The results with multiple imputation do not differ in significance or direction of significant associations of results without multiple imputation. Bold: p < 0.05.*

In addition to experiences of aggression, the use of force during the career appears to be significantly related to a higher population density in the work area, a higher rank and a higher risk for violence. Two-fifths of the variation in the use of force is explained by the measured characteristics (*R*^2^ not adjusted = 0.39). In addition to experiences of aggression, impatience with citizens is significantly related to the gender of police officers. Women appear to be more impatient with citizens than men. None of the other socio-demographic and work characteristics are associated with impatience with citizens. Overall, one ninth of the variation in impatience with citizens is explained by the measured characteristics (*R*^2^ not adjusted = 0.11).

**FIGURE 2:**
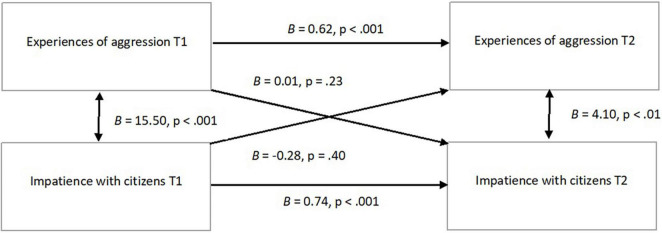
Cross lagged panel model of experiences of aggression and impatience with citizens, using FIML (*N* = 541).

Previous experiences of aggression are strongly related to experiences of aggression in the 6 months aftyerwards (*r* = 0.77, *p* < 0.001, not shown in the table) and earlier impatience with citizens is strongly related to impatience with citizens in the 6 months afterward (*r* = 0.68, *p* < 0.001, not shown in the table). These relationships are included in the next model. Figure 2 shows the cross-lagged panel model with which longitudinal effects of earlier experiences of aggression on later impatience and vice versa are tested. We thus focus on the diagonal paths in the figure. If these pathways were significant, this would be a strong indication of causality. However, this model shows that no relationship is found between earlier experiences of aggression and later impatience with citizens when taking into account earlier impatience with citizens and later experiences of aggression. It also shows that no relationship is found between earlier impatience with citizens and later experiences of aggression when earlier experiences of aggression and later impatience are taken into account. We therefore find no support for the presence of causal relationships between these variables, in either direction (not from earlier experiences of aggression to later measured impatience and not from earlier impatience to later measured experiences of aggression).

## Discussion

This study examined the extent to which police officers’ experiences of aggression are related to their behavior toward citizens, more specifically to the patience they have with citizens and the use of force weapons or tools in their career. In addition, two possible explanations for these potential relationships were examined, which emerged from the literature on victim-offender overlap (Jennings et al., 2012; Rokven, 2016). In this context, police officers are not seen as “perpetrators,” as they may and sometimes must use appropriate force in their job, so only the explanations for this phenomenon are used in this study. The first explanation from this literature, applied to the current research, assumes a causal relationship between the experiences of aggression and the behavior toward citizens. This can go in both directions: because of the aggression experience one becomes more impatient or because of the impatience one experiences aggression more often. The second explanation assumes that both the experiences of aggression and the behavior toward citizens are explained by other characteristics, for example the different situations in which the police officers find themselves during their work. This study examines for the first time to what extent these explanations are supported with regard to the relationship between police officers’ experiences of aggression and their behavior toward citizens.

First, the results of this study show that there is indeed a relationship between the experiences of aggression of police officers and the way they behave toward citizens. The more experiences of aggression, the more force the police officer uses on average during their career, and the more impatient police officers are with citizens. This is a strong and moderate relationship, respectively. A causal relationship between experiences of aggression and behavior toward citizens, which according to the first explanation should exist, was not be demonstrated in this study. The study does show, however, that the relationship is partly explained by other characteristics.

The results are partly in line with the formulated expectations, which were based on previous studies of aggression at work, including among police officers (Van Reemst et al., 2015; Ellrich and Baier, 2016; Noppe, 2018). For example, following those studies, a relationship was found between experiences of aggression, more use of force and impatience with citizens. It should be noted that the current study only concerns the use of force weapons and tools. Physical violence without using weapons or tools, which occurs more often among police officers than the use of the various weapons and tools (Kuin et al., 2020), was not included in this study. This means that the relationship between use of force and police officers’ experiences of aggression only concerns use of force weapons and tools. Whether the same results will apply to also physical violence should be studied in follow-up research.

The absence of direct support for the explanation that there is a causal relationship between behavior toward citizens and experiences of aggression differs from the expectations beforehand. It should be noted here that a definitive conclusion about a causal relationship on the basis of self-report in questionnaire research is actually not possible, so that is a limitation of this study. Drawing a definitive conclusion on causal relationship requires experimental or semi-experimental studies. Previous research on the victim offender overlap is often based on (observational) questionnaire research (Jennings et al., 2012) and has therefore not definitively established this causal relationship either. The analysis that has been done in this study, in which (subsequent) experiences of aggression are predicted on the basis of previous behavior toward citizens and previous experiences of aggression (and the same for behavior toward citizens), is also a very demanding test. For example, the earlier experiences of aggression appeared to be strongly related to the later experiences of aggression, leaving little room to demonstrate relationships with other variables. Therefore, the question is whether there really is no causal relationship or whether this has only not been demonstrated with the analysis in this study, for example because the time between the measurements was too short or too long. If there was a strong causal relationship, this would probably have become visible in the analysis, so the results do indicate that the direct effect of behavior toward citizens on experiences of aggression, and vice versa, is not very strong. Partial support is found for the other explanation, that the relationship between experiences of aggression and the behavior toward citizens is explained by other characteristics. Social demographic and work characteristics of the police officers appear to partly explain the relationship between experiences of aggression and behavior toward citizens. The fact that there is still a (smaller) relationship between experiences of aggression and the behavior toward citizens could mean that there is a direct link after all (cf. Rokven et al., 2013). However, it is also possible that all other characteristics that would explain the relationship have not yet been taken into account in this study.

Follow-up research could focus on these other characteristics that could further explain the relationship between experiences of aggression and behavior toward citizens, and were not included in this study. Based on the results of this study, it seems logical to focus mainly on characteristics related to situational risk of officers, as these characteristics partly explain the relationship between experiences of aggression and the behavior toward citizens. However, this study has already included characteristics that measure the situational risk of police officers on experiences of aggression. For this reason, one could also search for variables that provide a different explanation for the relationship, such as psychological characteristics of the officer, or norms of the organization and among colleagues about experiences of aggression and contact with citizens. Psychological characteristics may include sensation seeking or self-control. Self-control has been previously studied in the victim-offender overlap literature (Flexon et al., 2016). In Flexon’s study, it was found that, especially among men, self-control could explain a significant part of the relationship between perpetration and victimization. Future research should show whether this also applies to the relationship between behavior toward citizens and experiences of aggression among police officers. Moreover, this study on self-control gives reason to think that the explanations for a relationship between behavior toward citizens and experiences of aggression might differ between certain groups of police officers. Future research can look at this, for example by looking at differences between men and women (Flexon et al., 2016).

Further research is also needed into the causal relationship between behavior toward citizens and experiences of aggression. Experimental research on this subject, as with many outcome measures related to victimization and in criminology in general, is undesirable for ethical reasons. However, although advanced analysis techniques such as the cross-lagged panel model that was used in this study provide an approximation of a potential causal effect, these techniques also have limitations (Hamaker et al., 2015). Future research could therefore use other methods of data collection. Much research into this theme is now conducted on the basis of self-report questionnaire research. However, other types of data collection, such as the detailed analysis of video images in interactions between police officers and citizens, could perhaps provide more insight into the sequence of behavior of police officers and citizens and the links between them, as is already being done in other populations (Ejbye-Ernst et al., 2020).

This study has confirmed the relationship between behavior toward citizens and experiences of aggression for police officers in the Netherlands. In addition, the study contributed to identifying possible explanations for this relationship by applying the literature on the victim-offender overlap and testing the validity of these explanations. More detailed insight into this relationship is important for police practice and policy, for example to further promote positive interaction between police and citizens and ultimately to reduce the number of experiences of aggression among police officers, which is an aim in the Netherlands (Grapperhaus, 2021). It is already clear from the results of the current study that there is a relationship between experiencing aggression and behavior toward citizens, of which the police organization should be aware. Learning to deal with experiencing aggression can be included in police training. However, training and guidance during the career (for example during follow-up discussions and aftercare after aggression incidents and training related to aggression by citizens) is also important, and attention must be paid to experiences of aggression as well as to behavior toward citizens. Further research is needed to study the precise mechanisms of the relationship between behavior toward citizens and the experiences of aggression among police officers.

## Data Availability Statement

The data are not publicly available due to their containing information that could compromise the privacy of research participants. The data is available upon request to the corresponding author.

## Ethics Statement

Ethical review and approval was not required for the study on human participants in accordance with the local legislation and institutional requirements. The patients/participants provided their written informed consent to participate in this study.

## Author Contributions

LvR, TF, and FW contributed to conception and design of the study and wrote sections of the manuscript. LvR organized the database, performed the statistical analysis, and wrote the first draft of the manuscript. All authors contributed to manuscript revision, read, and approved the submitted version.

## Conflict of Interest

The authors declare that the research was conducted in the absence of any commercial or financial relationships that could be construed as a potential conflict of interest.

## Publisher’s Note

All claims expressed in this article are solely those of the authors and do not necessarily represent those of their affiliated organizations, or those of the publisher, the editors and the reviewers. Any product that may be evaluated in this article, or claim that may be made by its manufacturer, is not guaranteed or endorsed by the publisher.

## Footnotes

1 The term explanation is mentioned in this article because it is in line with the literature on this subject (Rokven et al., 2013). We note here that this is not truly a complete theoretical explanation, but rather a general theoretical perspective on how to explain why an empirical relationship is found between victimhood and perpetration.

2 Opposite effects also seem possible. After being a victim, someone might be at less risk of being a perpetrator, by behaving more withdrawn or becoming more patient. Or, because of being a perpetrator, one can have a higher risk of becoming a victim, for example because the behavior has a deterrent effect. However, no support for this is found in the literature and these are no explanations for the victim-offender overlap, because then there would be no overlap.

3 This was part of the first author’s dissertation. However, the research question and analyses in this article were not part of that thesis.

4 Data of the units obtained from the National Police.

5 The survey is composed of the scales that can be found in the references in this section.

6 These are not all questions of the Risk for Violence scale, because this scale was not specifically aimed at police officers, so it also contains questions about, for example, handling money during the job, which is a risk factor for violence in other professions, but does not apply among police officers. Those questions have therefore been omitted.

7 Because the questionnaire was already long, a number of possibly relevant variables were left out of the study. The study mainly focused on examining the role of individual psychological variables alongside situational characteristics that determine police work. We therefore did not include skills and in-group norms on aggression and behavior as these would lengthen the already significant questionnaire. No questions were asked about having participated in aggression training. Previous literature shows that the relationship with experiences of aggression is unclear. For example, it is regularly found that training is actually associated with more experiences of aggression, possibly because this type of training is more often offered or attended in areas where more aggression takes place (Fischer and Van Reemst, 2014).
